# Effects of *Clostridium butyricum* on Growth Performance, Health Status, and Metabolic Response of Pre-Weaning Angus Calves

**DOI:** 10.3390/ani15162332

**Published:** 2025-08-08

**Authors:** Jihong Wang, Xinze Yu, Yue Zhang, Yang Yang, Yukun Sun, Yonggen Zhang

**Affiliations:** College of Animal Science and Technology, Northeast Agricultural University, Harbin 150030, China; wjh19980422@outlook.com (J.W.); yuxinze0521@163.com (X.Y.); zhang_yue0930@163.com (Y.Z.); yangyang@neau.edu.cn (Y.Y.)

**Keywords:** *Clostridium butyricum*, calf, growth performance, health status

## Abstract

This study investigated the effects of *Clostridium butyricum* (*C. butyricum*) supplementation on the bacterial community composition and fermentation characteristics of the digestive system, assessed blood antioxidant capacity, and evaluated growth performance and digestibility in pre-weaning Angus calves. Sixteen male Angus calves were randomly divided into two groups (*n* = 8). The control group was fed a basal diet, while the other group was supplemented with 2 × 10^7^ colony-forming unit/d *C. butyricum* in the basal diet. After 8 weeks of feeding, body growth and nutrient intake were measured. Rumen fluid samples, blood samples, and fecal samples were collected and analyzed. Fecal scores were recorded daily, and the frequency of diarrhea treatments was documented. The results showed *C. butyricum* supplementation improved the digestibility of neutral detergent fiber, crude protein, and ether extract in calves, as well as the growth hormone content in the blood. Furthermore, supplementation elevated isovaleric acid concentrations in the rumen, modulated the microbiome, and increased pre-weaning body weight and average daily gain of calves. Meanwhile, *C. butyricum* supplementation reduced myeloperoxidase and superoxide dismutase levels while increasing total antioxidant capacity in the blood, alleviating oxidative stress, and enhancing the antioxidant capacity of the body. Intestinal health was also improved, as evidenced by an increase in butyrate content, a reduction in *enterotoxigenic Escherichia coli*, lower fecal scores, and a decreased need for diarrhea treatments. In summary, dietary *C. butyricum* supplementation may support digestive system development, enhance antioxidant capacity, and improve growth performance and health status in pre-weaning Angus calves.

## Simple Summary

As an effective feed strategy, long-term feeding of probiotics can affect the metabolism of the body and promote the growth performance and immune performance of livestock by regulating the digestive tract. The digestive system of pre-weaning calves gradually developed and was susceptible to external harmful bacteria that can disrupt the stability of the digestive tract microbiome, reduce immune performance, and lead to diarrhea. This study confirmed that long-term feeding of *Clostridium butyricum* can improve the gut microbiome of pre-weaning calves, enhance growth performance, improve antioxidant capacity, and reduce the occurrence of diarrhea.

## 1. Introduction

The pre-weaning period represents a critical phase in the growth and development of young ruminants, characterized by high morbidity and mortality rates [1]. This stage has a profound impact on the lifelong production performance and health of ruminants [2,3]. During this period, pre-weaning calves are susceptible to environmental stress, which can predispose them to diarrhea caused by various pathogenic and opportunistic bacteria [1]. The initiation of solid feed consumption plays a crucial role in stimulating digestive system development and stabilizing the gut microbiome [4,5]. In recent years, accumulating evidence has highlighted that administering appropriate doses of probiotic supplementations during the early stages of ruminants yields multiple benefits [6]. Probiotics aid in regulating the balance of the gastrointestinal microbiome, promoting host health and enhancing growth performance [6,7,8]. Therefore, utilizing the potential positive effects of probiotics to establish a protective microbiota in the gastrointestinal tract of calves and improve digestive system development and health is an effective feeding strategy.

During the fattening period, *Clostridium butyricum* (*C. butyricum*), as a probiotic, can effectively improve the rumen microbiome and enhance the growth performance of ruminants [9]. Additionally, *C. butyricum* has demonstrated growth-promoting effects in fattening goats under heat stress [10]. However, its impact on growth performance remains inconsistent, which may be related to the dosage of *C. butyricum* and the environment. Some studies report limited effects while highlighting its ability to enhance antioxidant capacity [11]. Regarding immune function, *C. butyricum* has been shown to modulate intestinal microbiome composition, activate immune factors, and improve intestinal immune function by preventing pathogen invasion [12,13,14].

*C. butyricum* has emerged as a promising feed additive option for disease prevention and treatment [15,16]. Butyric acid, as an important metabolite of *C. butyricum*, has been found to enhance digestive tract development in calves [17] and alleviate diarrhea [18]. Supplementation with sodium butyrate has been shown to enhance nutrient absorption in the rumen by increasing papillae length and volume [19,20]. Additionally, butyrate modulates the microbial environment and stimulating immune factors to prevent pathogen invasion, playing a crucial role in maintaining intestinal immune function [12,14]. Despite these potential benefits, the efficacy of *C. butyricum* as a probiotic in livestock remains inconsistent. One possible explanation is the disparity in the colonization ability of exogenous probiotics within the rumen or intestine before and after weaning, which feeding strategies and the internal physiological environment of the host may influence. Moreover, the specific effects of *C. butyricum* on the structural composition of the rumen and intestinal microbiome during calf development remain poorly understood, necessitating further investigation.

The present study aims to evaluate the effects of *C. butyricum* supplementation in pre-weaning calves. Specifically, this study examines its impact on growth performance, nutrient digestibility, the gastrointestinal environment, and microbial community composition. It is hypothesized that dietary *C. butyricum* supplementation will improve the gastrointestinal microbiome, enhance digestibility and production performance, and maintain body health in pre-weaning calves.

## 2. Materials and Methods

The study was conducted from April to July 2024 at a private ranch in Xing’an Prefecture, Inner Mongolia Autonomous Region, China. All experimental procedures were reviewed and approved by the Animal Care and Use Committee of Northeast Agricultural University, ensuring compliance with ethical standards for animal research (NEAUEC20240246). All calves were from the same herd.

### 2.1. Experimental Animals and Conditions

A total of 16 Angus calves (21 days old; mean body weight ± SD: 42.92 ± 2.70 kg) were enrolled in this randomized clinical trial. Inside the vacant, semi-open cowshed, there are 16 pens, each measuring 1.4 m × 1.4 m × 1.5 m, with ventilation fans installed on the ceiling. Immediately after birth, all calves were separated from their dams, weighed, and housed individually in pens with bedding, which was replaced every 24 h. According to the farm’s conventional calf-rearing protocol, a successful first feeding was defined as the consumption of at least 4 L of high-quality, treated colostrum (whiteness ≥ 22%, equivalent to immunoglobulin ≥ 50 mg/mL) within the first 4 h post-birth. Additionally, each calf received 2 L of colostrum at 12 h and again at 24 h post-birth. Milk replacer (MR) was administered at a rate of 4 L per day during the first week and increased to 6 L per day from the second week to the conclusion of the trial. All calves were bottle-fed throughout the study.

On the morning of day 21, body weights were recorded, and a randomized allocation sequence was generated using Microsoft Excel (Microsoft 365) based on body weight. Calves were then assigned to one of two groups (*n* = 8 per group): the control group (CON; received no compound probiotics supplementation) and the *C. butyricum* group (CB; received *C. butyricum* supplementation at a dose of 2 × 10^7^ CFU/d). Solid feed was provided after MR feeding every morning, and the feed composition is shown in Table 1. *C. butyricum* was applied by spraying it onto a small portion of the starter feed, which was provided first to ensure full probiotic consumption. The remaining starter feed was offered only after the initial portion was consumed. To maintain ad libitum access, at least 200 g of solid feed was left unconsumed daily. The study period spanned from day 21 to day 77, encompassing the pre-weaning phase. Following the conclusion of the experiment, a gradual weaning process was initiated from day 78 onward.

### 2.2. Measurement and Sample Collection

During the experimental period (days 21–77), individual body weights were assessed every 4 weeks. All body weight measurements were conducted in the morning before feeding using a digital scale (sensitivity = 0.1 kg), which had been inspected, certified, and validated beforehand. Based on the body weight measurements, the average daily weight gain of the two groups of animals was calculated. The feed conversion rate was determined by calculating the ratio of average daily feed intake to average daily weight gain.

Blood and rectal fecal samples were collected every 4 weeks. Blood samples were centrifuged at 3000× *g* for 10 min, and then 1 mL of serum/plasma was aliquoted and frozen at −20 °C or refrigerated at 4 °C for further processing. Approximately four hours after morning feeding, fresh fecal samples (5 g) were manually collected from the rectum of each calf and stored in sterile cryogenic vials at −80 °C. Additionally, approximately 2 g of fresh feces was placed in a plastic tube, acidified with 2 mL of 25% phosphoric acid, and diluted with 6 mL of distilled water. The mixture was centrifuged at 2500× *g* for 20 min at 4 °C, and the resulting supernatant was frozen at −20 °C for storage.

At 77 days, after a 3 h morning feeding, rumen fluid was collected using a flexible esophageal tube. Each collection was preceded by thorough rinsing with fresh water. The first 50 mL of collected rumen fluid was discarded to minimize saliva contamination. Subsequently, approximately 50 mL of rumen fluid (both solid and liquid components) was collected, and the pH value of the rumen fluid was immediately measured using a handheld electronic pH meter. Fresh rumen fluid was filtered through four layers of gauze, and a 5 mL sample of the filtered rumen fluid was transferred to a sterile cryogenic vial and stored at −80 °C. Additionally, 10 mL of rumen fluid was centrifuged at 2500× *g* for 20 min at 4 °C. After filtration, 1 mL of the supernatant was mixed with 0.25 mL of metaphosphoric acid standard solution (25 g/100 mL). The supernatant was stored at −20 °C for further laboratory analysis.

The fecal score was assessed every morning using Osorio’s criteria [21], with scores assigned as follows: a fecal score of 1 indicates a good fecal shape, 2 for soft (pudding-like) feces, 3 for wet (paste-like) feces, and 4 for liquid (spattered) feces. Scores 3 and 4 are considered diarrhea, and the diarrhea rate is calculated as the ratio of the number of diarrheic calves to the total number of calves. For calves with diarrhea, normal saline was administered intravenously, and Pulsatilla oral liquid and lactobacilli tablets were administered orally. All drugs are used under the guidance of the farm veterinarian. Daily medication records were kept, and medication was administered when calves exhibited diarrhea and fever.

### 2.3. Sample Analysis

Feed samples (Table 1) and fecal samples were collected weekly, dried at 55 °C for 48 h until a constant weight was achieved, ground, weighed, and sieved through a 1 mm sieve. Nutrient components, including dry matter (DM; method 925.40), crude protein (CP; method 955.04), and ether extract (EE; method 920.39), were collected according to validated methods described previously (AOAC, 2000) [22]. Acid Detergent Fiber (ADF; method 973.18) was determined according to the procedure of AOAC International (1990) [23]. NDF and ash (by combustion at 550 °C for 6 h) were calculated by following the procedure described by Goering and Van Soest [24]. All tests were repeated three times.

Oxidative biomarkers, including glutathione peroxidase, superoxide dismutase (SOD), myeloperoxidase (MPO), malondialdehyde (MDA), glutathione peroxidase (GSH-Px), and total antioxidant capacity (T-AOC), were analyzed according to the instructions provided in the respective reagent kits (Nanjing Jiancheng Bioengineering Research Institute Co., Ltd., Nanjing, China). Insulin-like growth factor-1 (IGF-1) was assayed using an ELISA kit and analyzed following the kit’s instructions (Nanjing Jiancheng Bioengineering Research Institute Co., Ltd., Nanjing, China). The enterotoxin gene content in feces was determined using an enterotoxigenic *Escherichia coli* PCR detection kit and analyzed based on the kit’s instructions (Shanghai Jonlnbio Industrial Co., Ltd., Shanghai, China).

Before analysis, fecal and rumen fluid supernatant samples were dissolved at room temperature. The supernatant was centrifuged at 13,000× *g* for 10 min at 4 °C, followed by VFA and NH_3_-N concentration analysis. The NH_3_-N in rumen fluid was determined using the alkaline hypochlorite phenol method and a spectrophotometer [25]. VFA concentrations were measured by gas chromatography equipped with a hydrogen flame detector and a capillary column (Agilent Technologies, Santa Clara, CA, USA; length 30 m, diameter 0.32 mm, membrane thickness 0.50 μm), following the procedures described by Hu et al. [26].

Microbiological analysis was conducted on the fecal samples and rumen fluid from both groups. DNA was extracted from the samples following the instructions provided by the DNA extraction kit (Omega Bio-Tek, Norcross, GA, USA). The concentration and purity of DNA were measured at 260 and 280 nm using a spectrophotometer (UV-1700, Shimadzu Corporation, Kyoto, Japan). PCR amplification and 16S rRNA sequencing were performed according to the procedure described by Zhou et al. [27]. Subsequently, paired-end sequencing was performed on the community DNA fragments from the fecal and rumen fluid samples using the Illumina platform. The raw sequencing data were stored in FASTQ format.

### 2.4. Sample Analysis

Except for microbial flora data, all results were taken as the mean after three repetitions, and all statistical analyses were conducted using SPSS 27.0 software (SPSS Inc., Chicago, IL, USA). Treatment efficacy was estimated using the terminal body weight through the efficacy analysis model program. The normality of the data was assessed using the Shapiro–Wilk test, and the data excluding the microbial flora were found to be normally distributed (*p* > 0.05). The mixed linear model program was used to analyze the effects of calves as a random effect and initial body weight as a fixed effect on growth performance, and the effects were negligible (*p* > 0.05). Other data were also analyzed using the mixed linear model program to assess the impact of randomized blocks on indicators, but the impact was negligible (*p* > 0.05). Continuous variables were analyzed using One-way ANOVA, and significant differences were tested using the Tukey method. *p* < 0.05 indicated significant differences, and *p* ≤ 0.01 indicated extremely significant differences. For the microbial sequences saved in FASTQ format, Microbioanalysis 2.0 was used for data processing, analysis (e.g., PCA, PLS-DA, orthogonal partial least square discriminant analysis [OPLS-DA], the nonparametric Kruskal–Wallis test, the Student’s *t*-test, the Mann–Whitney–Wilcoxon U test, PERMANOVA, and correlation analysis), calibration (e.g., FDR), and visualization of characteristic microbial groups. Data visualization was performed using the GraphPad Prism 9.5 software package (GraphPad Inc., La Jolla, CA, USA).

## 3. Results

### 3.1. The Impact of C. butyricum on the Growth and Digestive Performance of Calves

Supplementation with *C. butyricum* increased pre-weaning daily weight gain by 15.0%, leading to a significant 16.6% increase in body weight at 77 days of age (Table 2). The power calculated by One-way ANOVA analysis based on the final body weight of the experiment was 0.77. Although *C. butyricum* supplementation resulted in a slight increase in average dry matter intake, it did not significantly affect feed efficiency (*p* > 0.05; Table 3). There were no significant differences in dry matter and ADF digestibility (*p* > 0.05). However, compared to the CON group, the CB group exhibited significant improvements in nutrient digestibility, with increases of 7% in NDF digestibility (*p* = 0.03), 15.4% in apparent CP digestibility (*p* = 0.01), and 17.4% in apparent EE digestibility (*p* = 0.01).

### 3.2. The Impact of C. butyricum on Rumen Fermentation and Microbial Community in Calves

The effects of *C. butyricum* supplementation on rumen fermentation parameters are shown in Table 4. The pH values in the rumen of the two groups were basically consistent (*p* > 0.05). Additionally, no significant differences were observed in total volatile fatty acid concentration or the concentrations of acetic acid, propionic acid, butyric acid, and valeric acid (*p* > 0.05). However, compared to the CON group, the calves in the CB group exhibited a 55.4% increase in isovaleric acid content in the rumen (*p* = 0.05) and a twofold increase in NH3-N content (*p* < 0.01)

**Figure 1:**
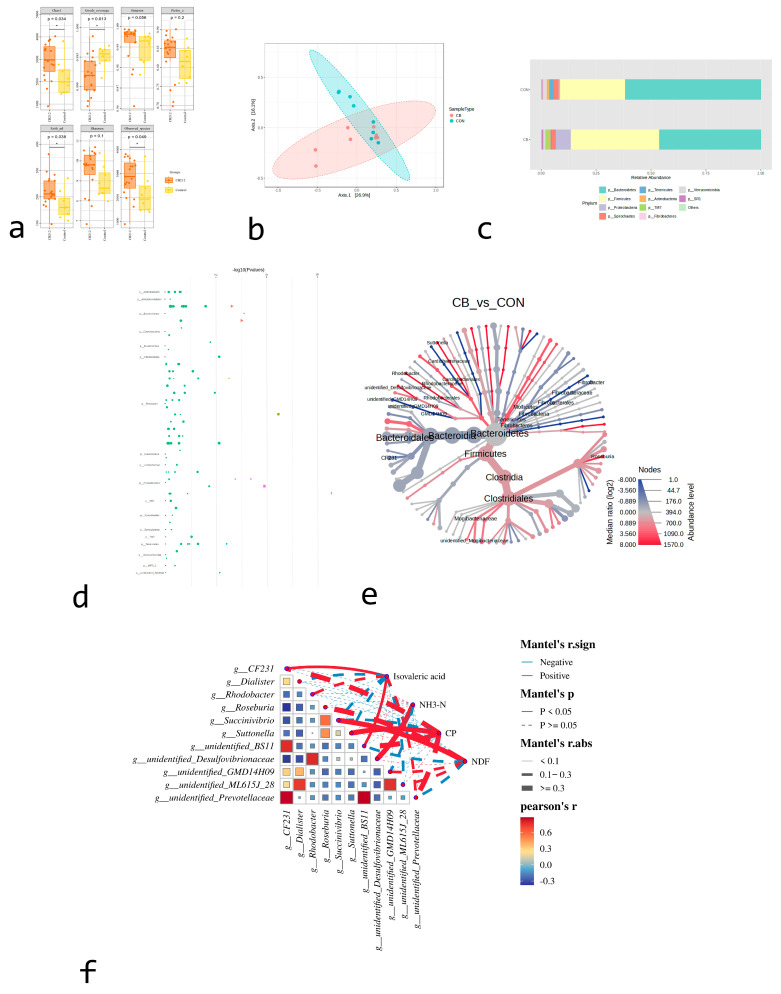
Effects of addition of *C. butyricum* (CB; *n* = 7) or not (CON; *n* = 7) on bacterial flora in rumen of pre-weaning calves. (**a**) Shannon index, observed species, Chao1, and Simpson index α-diversity between groups; means ± SEM. (**b**) Principal coordinate analysis (PCoA) of rumen bacteria based on weighted UniFrac distance matrices. (**c**) Relative abundance of top-10 bacteria in rumen of pre-weaning calves. (**d**) Cluster analysis heatmap of 11 genera with differences between groups. (**e**) Clustering-based tree diagram of 11 genera with differences between groups. (**f**) Correlation analysis diagram of isovaleric acid, NH_3_-N, CP, and NDF with differential genera. Correspondence between color gradients and values is indicated in gradient color blocks. Genera variation is represented by Z-Score.

Alpha diversity analysis was conducted using Shannon index, observed species, Chao1, and Simpson index; the results showed that compared with the rumen microbiome composition in the CON group, there were more diverse and distinct bacterial communities in the CB group (Figure 1a). Beta diversity analysis was conducted using nonparametric permutational multivariate ANOVA (PERMANOVA) tests with 999 permutations to measure the compositional similarities between bacterial communities within groups of samples. For this, abundance data based on weighted UniFrac distance matrices were analyzed. The results showed that significant divergences existed between the groups (Figure 1b).

A total of 14 phyla were identified in each group. In the CON group, *Bacteroidetes* was the dominant phylum. Furthermore, compared to the CON group, the CB group exhibited enrichment in *Firmicutes* (Figure 1c). To compare the differences in fecal microbiome between the two groups, Mann–Whitney U tests were conducted at different taxonomic levels. At the genus level, significant differences were observed in 11 genera between the two groups (Figure 1d). Specifically, the relative abundances of *CF231* and *Dialister* decreased in the CB group, while the relative abundances of *Rhodobacter*, *Roseburia*, *Succinivibrio*, and *Suttonella* increased in the CB group. Cluster analysis of the differentially expressed bacterial genera is shown in Figure 1e, while Figure 1f presents the correlation between these genera and ruminal isovaleric acid and NH_3_-N concentrations.

### 3.3. The Effects of C. butyricum on Blood Glucose, Blood Ketone, Blood Antioxidant Capacity, and Hormones in Calves

The effects of experimental treatments on antioxidant capacity and hormone levels in pre-weaning calves are summarized in Table 5. Supplementation with *C. butyricum* increased the content of IGF-1 in the blood (*p* = 0.05). Additionally, *C. butyricum* supplementation significantly reduced T-SOD activity in the blood (*p* = 0.01) but had no significant effect on malondialdehyde (MDA) or glutathione peroxidase (GSH-Px) concentrations (*p* > 0.05). However, MPO concentration was reduced by 14.4% (*p* = 0.04), which subsequently led to a significant 16.2% increase in T-AOC (*p* < 0.01).

### 3.4. The Effects of C. butyricum on Intestinal Health and Fecal Characteristics in Calves

The effects of *C. butyricum* supplementation on fecal characteristics, diarrhea incidence, intestinal butyrate levels, and MPO content are presented in Table 6. Compared with the CON group, *C. butyricum* supplementation significantly increased intestinal butyrate concentration by 56.9% (*p* = 0.02), while MPO content was markedly reduced by 61.8% (*p* = 0.04). Consistently, fecal scores were significantly lower in the CB group (*p* = 0.02), indicating improved stool consistency, and the frequency of diarrhea treatment was reduced. Further assessment of enterotoxin-producing genes revealed a lower number of positive samples in the CB group.

**Figure 2:**
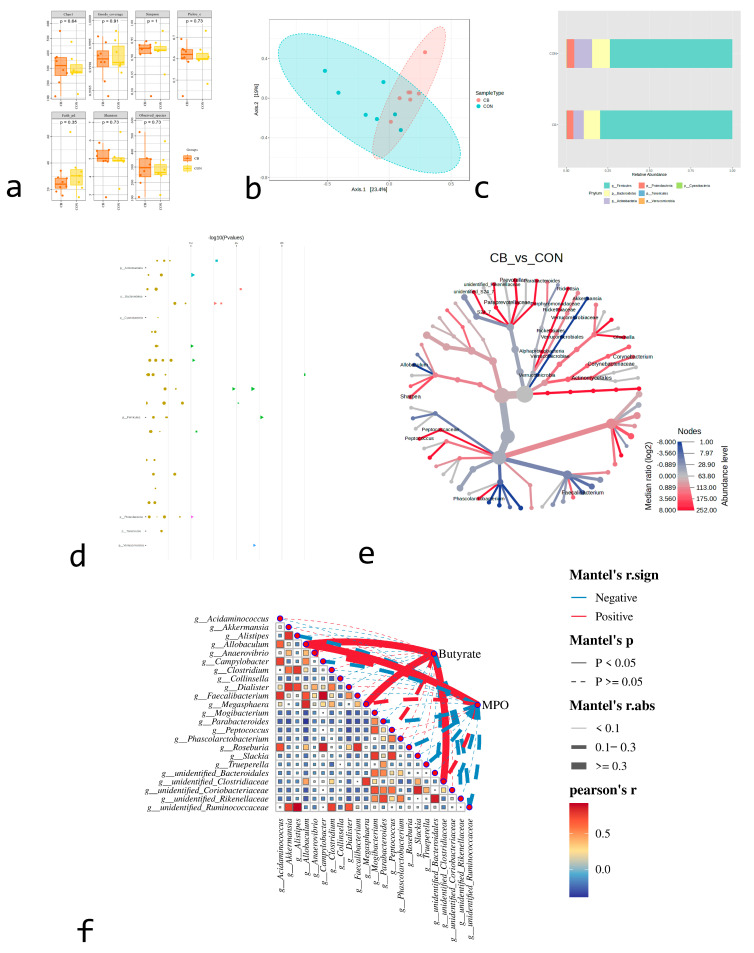
Effects of adding *C. butyricum* (CB; *n* = 7) or not (CON; *n* = 7) on bacterial flora in feces of pre-weaning calves. (**a**) Alpha diversity index based on Shannon index, observed species, Chao1, and Simpson between groups; means ± SEM. (**b**) Fecal bacterial principal coordinate analysis (PCoA). (**c**) Relative abundance of seven bacteria in feces of pre-weaning calves. (**d**) Cluster analysis heatmap of 19 genera with differences between groups. (**e**) Clustering-based tree diagram of 19 genera with differences between groups. (**f**) Correlation analysis diagram of butyric acid and MPO with 19 differentially expressed genera. Colors represent relative abundance of genera in sample group, and correspondence between color gradients and values is indicated in gradient color blocks. Genera variation is represented by Z-Score.

Multiple analysis methods were used to measure the alpha diversity index, and the results showed no significant difference in the total microbial content of the fecal microbiome between the two groups (Figure 2a). PERMANOVA tests were used to analyze the beta diversity of abundance data based on a weighted UniFrac distance matrix. The results showed that there were significant differences among the groups (Figure 2b).

A total of seven phyla were identified in each group. *Proteobacteria* exhibited a relative enrichment in the CB group compared to the CON group (8.67% vs. 4.36%) (Figure 2c). To compare the differences in fecal microbiome between the two groups, Mann–Whitney U tests were conducted at different taxonomic levels. At the genus level, significant differences were observed in 19 genera between the two groups. Compared to the CON group, the relative abundances of *Akkermansia*, *Alistipes*, *Allobaculum*, *Anaerovibrio*, *Collinsella*, *Dialister*, *Phascolarctobacterium*, *Roseburia*, and *Sutterella* decreased in the CB group, while the relative abundances of *Desulfovibrio*, *Faecalibacterium*, *Megasphaera*, *Parabacteroides*, *Peptococcus*, and *Slackia increased* (Figure 2d). Cluster analysis of these differentially expressed bacterial genera is illustrated in Figure 2e, while Figure 2f presents the correlation analysis between these genera and intestinal butyrate and MPO levels.

## 4. Discussion

Our findings demonstrate that *C. butyricum* supplementation significantly enhanced daily weight gain, consistent with previous studies reporting improved similar effects [9,11]. *C. butyricum* dietary supplementation significantly improved growth performance but had no significant effect on feed conversion efficiency [9]. However, other studies have reported no significant improvements in growth performance following *C. butyricum* supplementation in ruminants [28]. For instance, Zhang found that daily supplementation with *C. butyricum* at doses of 5 × 10^9^ CFU or 2 × 10^10^ CFU had no significant effect on the growth performance and apparent digestibility of fattening goats [11]. The variability in response to *C. butyricum* supplementation may be attributed to differences in dosage [6]. Supporting this, Cai et al. and Xue et al. reported that while lower doses of *C. butyricum* (2 × 10^8^ CFU and 1 × 10^9^ CFU per day) did not significantly enhance weight gain in fattening goats, higher doses improved daily weight gain by 24.7% [10,29]. Additionally, although there are no published reports on the differences in the effects of strain differences on the growth performance of ruminants, there are significant differences in the effects of different strains on the body. Different strains of *Lactobacillus acidophilus* at the same therapeutic dose have different therapeutic effects on patients with irritable bowel syndrome. Oral administration of 1 × 10^10^ CFU of *Lactobacillus acidophilus DDS-1* per day effectively improves the severity of irritable bowel syndrome symptoms, while the same dose of *Lactobacillus acidophilus NCFM* has no significant effect [30,31].

Overall, the addition of *C. butyricum* improved the digestibility of nutrients in the rumen. Compared to the control group, calves receiving 2 × 10^7^ CFU/day of *C. butyricum* exhibited improved digestibility of NDF [32], crude fat, and crude protein. Cai et al. found that daily supplementation of 1.3 × 10^7^ CFU and 2.6 × 10^7^ CFU of *C. butyricum* effectively improved the utilization of DM, NDF, and ADF by the goat digestive system and effectively increased dry matter intake and daily gain [10]. Many researchers have demonstrated that butyric acid provides energy for absorption in the rumen epithelium or increases blood flow out of the rumen, thereby improving the diffusion rate of VFA and promoting the integrity of the rumen epithelium [32,33]. This may be the reason for the higher concentration of ammonia nitrogen in the rumen of the CB group. Furthermore, it enhances rumen microbial protein synthesis or nitrogen utilization, thereby effectively improving protein utilization efficiency. The rapid diffusion of VFA improves the digestibility of nutrients, thereby enhancing growth performance.

To further demonstrate the impact of *C. butyricum* on the development of the rumen in calves, this study observed the differences in the rumen microbiome. Overall, *C. butyricum* supplementation effectively enhanced microbial richness and altered the microbial community structure. Notably, feeding *C. butyricum* significantly increased the relative abundance of the fiber-degrading bacterium genus *Succinivibrio* [34], as well as the relative abundances of species involved in biohydrogenation such as *Roseburia* [35], *Rhodobacter*, and *Suttonella* [36], which are associated with body weight gain. Meanwhile, the relative abundances of *CF231* and *Dialister* decreased. These findings contrast with previous reports indicating that probiotic supplementation can enrich *CF231* in the rumen and enhance daily weight gain in lambs [37]. This may be due to the higher abundance of microbial communities in the CB group, where the content of the *CF231* genus increased, but its relative abundance decreased. Through a joint analysis of NDF and CP digestibility, as well as rumen differential metabolites and differential microbiomes, we found that isovaleric acid content increased and positively correlated with changes in *CF231*. Additionally, the relative abundance of *Succinivibrio*, a key fiber-degrading bacterium, was elevated, leading to improved apparent digestibility of NDF [34]. Similarly, apparent CP digestibility was positively correlated with the relative abundance of *Suttonella*, which has also been linked to enhanced body weight gain [36]. These findings indicate that *C. butyricum* improves rumen fermentation efficiency and microbial balance, thereby enhancing digestibility and growth performance in pre-weaning calves.

*C. butyricum* supplementation also influences oxidative biomarkers, which serve as indicators of physiological and health status [38]. Butyric acid, as an important metabolite of *C. butyricum*, significantly increases the activity of the antioxidant enzyme SOD, reduces the concentration of lipid peroxidation product MDA, and enhances the antioxidant capacity of calves when administered as a sole supplement [39]. Similar effects have been reported in monogastric animals, where *C. butyricum* supplementation significantly increased antioxidant enzyme activity, reduced oxidative stress markers, and improved total antioxidant capacity (T-AOC) [14,40]. However, studies on ruminants have shown that feeding *C. butyricum* has no significant effect on blood antioxidant capacity [9]. In this study, daily supplementation with 2 × 10^7^ CFU of *C. butyricum* significantly reduced total SOD (T-SOD) activity while markedly enhancing T-AOC activity. This implies that feeding *C. butyricum* reduces the activity of some antioxidant enzymes in the body but increases the expression of other antioxidant pathways, thereby enhancing antioxidant capacity. MPO, a key oxidative stress marker involved in neutrophil-mediated immune responses [41], was significantly reduced following *C. butyricum* supplementation. This decrease in MPO levels further supports the hypothesis that *C. butyricum* mitigates oxidative stress and inflammation in pre-weaning calves. Collectively, these findings indicate that incorporating *C. butyricum* into starter feed enhances the antioxidant capacity and immune function of calves while decreasing the frequency of diarrhea treatments.

Pre-weaning calves are particularly prone to gastrointestinal diseases, which can affect their growth performance. However, *C. butyricum* has been shown to improve intestinal flora and enhance intestinal health [14,42]. This study also found that feeding *C. butyricum* can effectively reduce the frequency of drug treatment and the rate of diarrhea. Butyrate in the calf’s intestine can affect histone acetylation, which in turn affects gene expression, preventing the colonization of harmful pathogens in the gastrointestinal tract, and is positively correlated with intestinal health [17]. Additionally, butyric acid-producing genera, such as *Faecalibacterium* and *Allobaculum*, exhibited increased relative abundances in the CB-supplemented group [43,44], which has the function of reducing the production of inflammatory factors [45]. The positive correlation observed between intestinal butyric acid content and *Allobaculum* abundance in this study further supports the hypothesis that *C. butyricum* enhances gut health by promoting the proliferation of beneficial butyrate-producing bacteria. *Parabacteroides* has also been shown to have probiotic and significant anti-inflammatory effects [46]. The relative abundance of *Faecalibacterium* and *Parabacteroides* significantly increased in the intestines of calves supplemented with *C. butyricum*, but contrary to the results of Zhu, *C. butyricum* also reduced the abundance of *Alistipes* and *Anaerovibrio* genera [47,48]. Interestingly, *Megasphaera* abundance has been reported to increase following oral administration of *Escherichia coli K88*; however, in this study, *C. butyricum* supplementation effectively reduced *Megasphaera* levels, contradicting previous findings [49]. Conversely, another study found that *C. butyricum* supplementation increased *Megasphaera* abundance, highlighting its potential role in gut health regulation [15]. Meanwhile, *Collinsella*, which has been associated with increased infection susceptibility [50], exhibited a decline in relative abundance following *C. butyricum* supplementation. Although no significant difference was observed in the overall abundance of *Escherichia coli* between groups, fewer fecal samples tested positive for enterotoxin-producing *Escherichia coli* in the CB group. This suggests a potential reduction in pathogenic *Escherichia coli* populations. The content of MPO in feces was significantly correlated with the content of enterotoxin-producing *Escherichia coli* [40]. These findings further validate the ability of *C. butyricum* to mitigate enterotoxin-producing Escherichia coli populations in the gut. Collectively, this study demonstrates that *C. butyricum* supplementation modulates gut microbiome composition, increases butyric acid production, and reduces pathogenic and opportunistic bacteria. These changes suggest its potential to improve gut health.

## 5. Conclusions

Overall, *C. butyricum* supplementation enhances rumen fermentation and elevates the relative abundances of species involved in biohydrogenation and fiber degradation, improving nutrient digestibility in pre-weaned calves. Additionally, *C. butyricum* supplementation enhances the relative abundance of butyric acid-producing genera and butyrate levels in the gut, decreases the presence of harmful bacteria, and bolsters antioxidant defenses and immune functions, ultimately enhancing calf growth performance. These results demonstrate the biological significance of supplementing *C. butyricum* in early calf feeding in livestock systems. Furthermore, the impact of these results on post-weaning calves warrants further investigation.

## Figures and Tables

**Table 1:** Nutrient composition analysis of grass hay, calf starter, and milk replacer on DM basis.

Items	Calf Starter	Grass Hay	Milk Replacer
DM, %	90.07	97.79	91.85
CP, %	20.18	8.48	22.39
EE, %	3.96	8.04	15.62
Ash, %	7.89	6.04	7.94
NDF, %	29.18	40.76	
ADF, %	10.38	39.12	

**Table 2:** Effects of *C. butyricum* on growth performance and feed intake of pre-weaning calves ^1^.

	Groups			
Items	CON	CB	SEM	*p*-Value
Initial weight, kg	42.53	43.29	0.68	0.61
Weaning weight, kg	94.06 b	109.54 a	3.12	0.01
DMI ^2^, kg/d	1.93	2.20	0.08	0.11
ADG, kg/d	1.00 b	1.15 a	0.04	0.03
FE ^3^	2.01	1.99	0.07	0.24

^1^ CON = control dietary treatment (*n* = 8), and CB = dietary treatment supplemented with 2 × 10^7^ CFU *C. butyricum*/day (*n* = 8). During the 56-day experimental period, *C. butyricum* was directly fed with a solid diet as a carrier. ^2^ DMI = total DM of feed, including milk replacer, grass hay, and starter pellets. ^3^ FE = feed efficiency, the ratio of DMI to ADG. a, b, indicate significant differences (*p* < 0.05).

**Table 3:** Effects of *C. butyricum* on digestibility of pre-weaning calves ^1^.

	Groups			
Items	CON	CB	SEM	*p*-Value
DM, %	93.18	93.18	0.18	0.99
NDF, % of DM	59.62 b	63.82 a	1.46	0.03
ADF, % of DM	46.04	46.48	0.83	0.80
EE, % of DM	66.78 b	77.06 a	1.85	0.01
CP, % of DM	50.56 b	59.35 a	1.82	0.01

^1^ CON = control dietary treatment (*n* = 8), and CB = dietary treatment supplemented with 2 × 10^7^ CFU *C. butyricum*/day (*n* = 8). During the 56-day experimental period, *C. butyricum* was directly fed with a solid diet as a carrier. The digestibility was determined with acid-insoluble ash as an internal reference. a, b, indicate significant differences (*p* < 0.05).

**Table 4:** Effects of *C. butyricum* on ruminal fermentation parameters of pre-weaning calves ^1^.

	Groups			
Items	CON	CB	SEM	*p*-Value
pH	6.14	5.96	0.16	0.59
Acetic acid, mmol/L	51.93	42.28	5.16	0.37
Propionic acid, mmol/L	17.99	18.26	1.79	0.85
Butyric acid, mmol/L	8.73	9.81	1.23	0.80
Isovaleric acid, mmol/L	0.65 b	1.01 a	0.09	0.05
Valeric acid, mmol/L	1.30	1.31	0.12	0.96
Total VFA, mmol/L	81.60	66.16	6.18	0.23
NH3-N, mmol/L	4.08 b	12.37 a	1.37	0.01

^1^ CON = control dietary treatment (*n* = 8), and CB = dietary treatment supplemented with 2 × 10^7^ CFU *C. butyricum*/day (*n* = 8). During the 56-day experimental period, *C. butyricum* was directly fed with a solid diet as a carrier. a, b, indicate significant differences (*p* < 0.05).

**Table 5:** Effects of *C. butyricum* on blood glucose, blood ketones, blood antioxidant activity, and hormones of pre-weaning calves ^1^.

	Groups			
Items	CON	CB	SEM	*p*-Value
IGF-1, ng/mL	2.13 b	7.45 a	1.37	0.05
MPO, U/mL	27.29 a	23.35 b	0.99	0.04
T-SOD, U/mL	4.07 a	3.01 b	0.23	0.01
GSH-Px, U/mL	171.59	171.97	14.23	0.99
T-AOC, U/mL	10.34	12.01	0.34	<0.01
MDA, nmo/mL	3.01	2.87	0.18	0.71
blood glucose, mmo/L	5.48	4.81	0.30	0.27
ꞵ-hydroxybutyric acid, mmo/L	0.24	0.26	0.04	0.85

^1^ CON = control dietary treatment (*n* = 8), and CB = dietary treatment supplemented with 2 × 10^7^ CFU *C. butyricum*/day (*n* = 8). During the 56-day experimental period, *C. butyricum* was directly fed with a solid diet as a carrier. a, b, indicate significant differences (*p* < 0.05).

**Table 6:** Effects of *C. butyricum* on butyric acid content, MPO levels, fecal scores, diarrhea frequency, and presence of enterotoxigenic genes of pre-weaning calves ^1^.

	Groups			
Items	CON	CB	SEM	*p*-Value
Butyric acid, mmol/L	0.51 b	0.80 a	0.06	0.02
MPO, U/g	2.83	1.08	0.44	0.04
Fecal score	1.19 a	1.04 b	0.03	0.02
Diarrhea ^2^	9	2		
Positive sample ^3^	7	3		

^1^ CON = control dietary treatment (*n* = 8), and CB = dietary treatment supplemented with 2 × 10^7^ CFU *C. butyricum*/day (*n* = 8). During the 56-day experimental period, *C. butyricum* was directly fed with a solid diet as a carrier. ^2^ Diarrhea = number of treatments for calves with fecal scores ≥ 3. ^3^ Positive sample = number of positive samples for enterotoxin gene detection in feces. a, b, indicate significant differences (*p* < 0.05).

## Data Availability

The raw data supporting the conclusions of this article will be made available by the authors on request.

## Abbreviations

The following abbreviations are used in this manuscript:

*C. butyricum*	*Clostridium butyricum*
CFU	colony-forming unit
DMI	dry matter of feed
CP	crude protein
EE	ether extract
NDF	neutral detergent fiber
ADF	acid detergent fiber
ADG	average daily gain
MPO	myeloperoxidase
SOD	superoxide dismutase
T-AOC	total antioxidant capacity
VFA	volatile fatty acid
NH_3_-N	ammoniacal nitrogen
MDA	malondialdehyde
GSH-Px	glutathione peroxidase
IGF-1	Insulin-like growth factor-1
